# Manual of Operative Surgery

**Published:** 1886-10

**Authors:** 


MANUAL OF OPERATIVE SURGERY. *By* LEWIS A. STIMSON, B. A., M. D., *Surgeon to the Presbyterian and Bellevue Hospitals, Professor of Clinical Surgery in the Medical Faculty of the University of the City of New York, etc. Second Edition, with 342 illustrations, pp. 506. Philadelphia:* LEA BROTHERS & CO. *Chicago:* JANSEN, McCLURG & CO.

In this interesting and practical work, which is in its second edition, the principal changes from the former edition will be found, as the author states in his preface, "in the manner of operative procedures brought about by the antiseptic method of treating wounds, and of excision of bones and joints, and operations in which the peritoneal cavity is opened." The directions for operations are clear and concise, and sufficiently minute in details and in the regional anatomy of the parts, to be easily understood by all who have a reasonable knowledge of the subject.

The illustrations, which are three hundred and forty-two in number, are especially worthy of note, as they are well executed, and many times make a more clear and lasting impression on the mind than could be gained by a study of the text alone. The typographical work in the book is very good. F. W. C.

